# Safety and efficacy of a new tourniquet system

**DOI:** 10.1186/1471-2482-12-17

**Published:** 2012-08-15

**Authors:** Junko Sato, Yoshinori Ishii, Hideo Noguchi, Mitsuhiro Takeda

**Affiliations:** 1 Ishii Orthopaedic & Rehabilitation clinic, 1089 Shimo-oshi, Saitama, Japan

**Keywords:** Tourniquet, Upper limb surgery, Systolic blood pressure, Autoregulation

## Abstract

**Background:**

In upper limb surgery, the pneumatic tourniquet is an essential tool to provide a clean, bloodless surgical field, improving visualization of anatomical structures and preventing iatrogenic failure. Optimal inflation pressure to accomplish these objects without injuring normal tissue and inducing complications is not yet established. Use of the minimum tourniquet pressure necessary to produce a bloodless surgical field is preferable in order to prevent injury to normal tissue. Various methods have been implemented in an effort to lower effective cuff pressure. The purpose of this study is to report clinical experience with a new tourniquet system in which pressure is synchronized with systolic blood pressure (SBP) using a vital information monitor.

**Methods:**

We routinely used the tourniquet system in 120 consecutive upper limb surgeries performed under general anaesthesia in our operating room instead of our clinic. Cuff pressure was automatically regulated to additional 100 mmHg based on the SBP and was renewed every 2.5 minutes intervals.

**Results:**

An excellent bloodless field was obtained in 119 cases, with the exception of one case of a 44-year-old woman who underwent internal screw fixation of metacarpal fracture. No complications, such as compartment syndrome, deep vein disorder, skin disorder, paresis, or nerve damage, occurred during or after surgery.

**Conclusions:**

This new tourniquet system, synchronized with SBP, can be varied to correspond with sharp rises or drops in SBP to supply adequate pressure. The system reduces labor needed to deflate and re-inflate to achieve different pressures. It also seemed to contribute to the safety in upper limb surgery, in spite of rare unexpected oozing mid-surgery, by reducing tissue pressure.

## Background

In upper limb surgery, the pneumatic tourniquet is an essential tool to provide a clean, bloodless surgical field, which improves visualization of anatomical structures and prevents iatrogenic failure. Optimal inflation pressure to accomplish these objects without injuring normal tissue is not yet established. Severe complications are very rare with the use of conventional pressure-retained type tourniquets in upper limb surgery [1-3]. However, temporary or irreversible tissue damage may occur even with appropriate tourniquet usage [1,2]. Most cases of neurologic complications are due to excessively high inflation pressure or long period of inflation [1-5]. Graham et al. [6] used a fully implantable biomedical pressure transducer with cadaver upper extremities to demonstrate that tourniquet cuffs transmit high pressures to peripheral nerves under its midpoint without significant attenuation by the intervening soft tissues in an upper arm; he also reported a steep gradient of perineural pressure that decreased near the locations beneath the edge of the cuff. These results suggest the use of lower inflation pressure whenever possible.

Conventional tourniquet systems remain on the initial setting pressure throughout the procedure. However, blood pressure can vary during surgery, and conventional systems cannot respond to sharp blood pressure changes. This might result in additional time used to manually adjust pressure, or contaminating the dry surgical field and promoting tissue edema by oozing. Ishii et al. [7] previously advocated the usefulness of a new tourniquet system for foot and ankle surgery with tourniquet inflation pressure in synchrony with systolic blood pressure (SBP) to maintain a bloodless surgical field. The current study reports the results of clinical application of this new tourniquet system for upper limb surgery.

## Methods

### Usage of the tourniquet

**Figure 1:**
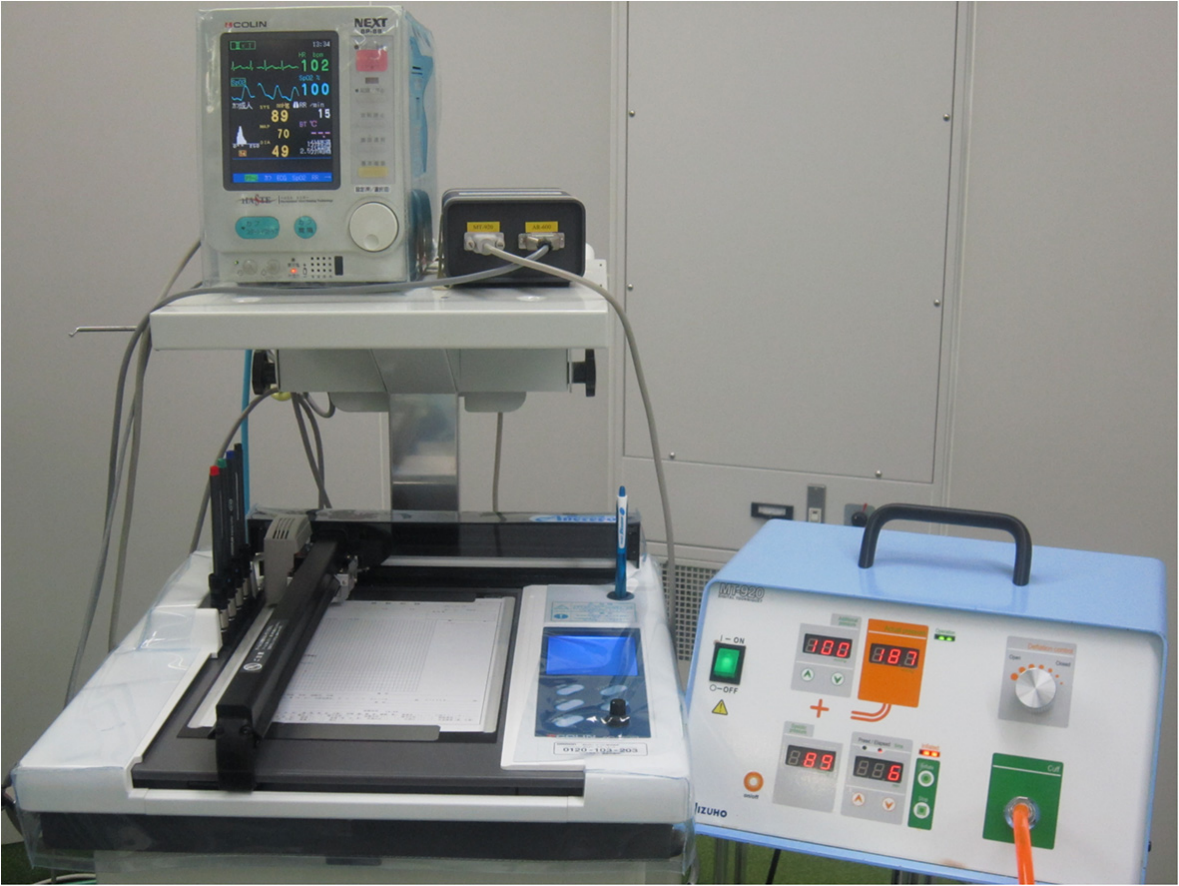
**The MT-920 tourniquet system (Mizuho-Ika, Tokyo, Japan) (*right*) and the vital information monitor (*left*).**

**Figure 2:**
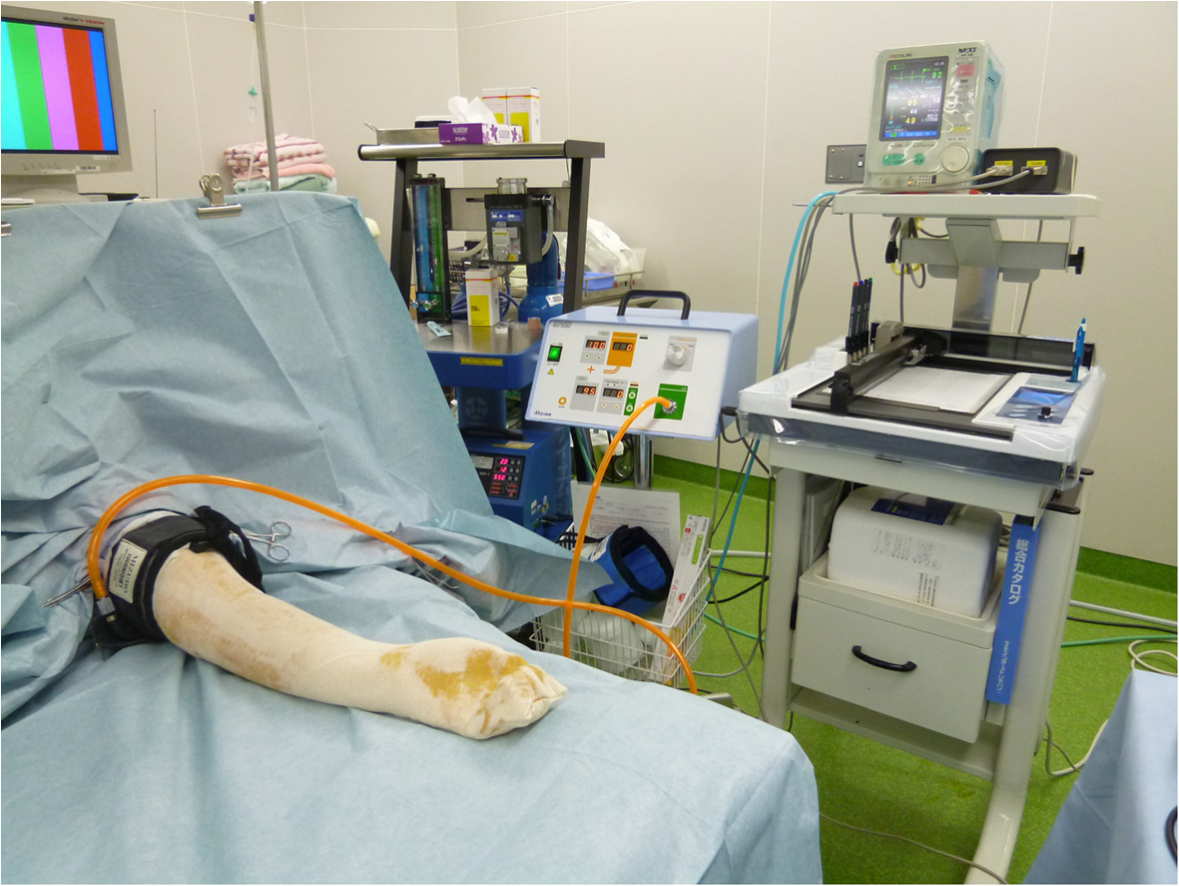
Illustration showing the use of the MT-920 tourniquet system for the surgery of distal radius fracture.

**Figure 3:**
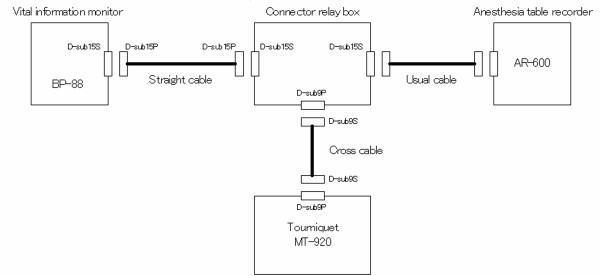
Scheme of the MT-920 tourniquet system.

We used a new tourniquet system in which pressure is synchronized with SBP, using a vital information monitor. The MT-920 tourniquet system (Mizuho-Ika, Tokyo, Japan) constantly reads monitored data, and operates in real-time with a change in blood pressure [7] (Figure 1-2). This system renews inflated pressure automatically based on SBP at predetermined intervals (Figure 3). The vital information monitor, BP-88, adopts a simple arm cuff since this system is a cuff sphygmomanometer. The blood pressure of a patient is monitored and the MT-920 system acquires the information with a connector broadcast box (connector relay box) since it can be transmitted as a digital signal. The information is also recorded by the anaesthesia notation record device AR-600. The timing and frequency of the measurement are controlled by the BP-88. The data are always updated when each measurement is finished. Since the MT-920 system constantly reads data, it operates in real-time with a change in the blood pressure.

We routinely applied additional pressure of 100 mmHg based on the SBP and 2.5 minutes as the renewing interval. After exsanguination of the upper limb by elevation and an Escmarch bandage, tourniquet was inflated initially before skin incision. After beginning the operation, the actual pressure produced with this system was within 10 mmHg of the desired pressure. The tourniquet cuff was 88 cm long and 8.5 cm wide for adults, and 68 cm long and 7 cm wide for children. Four or five layers of cast padding were applied under the cuff to protect the skin. The tourniquet was positioned on the upper arm in all cases. The same surgical team performed all operations in a laminar-flow operating room.

### Subjects

One-hundred twenty patients, scheduled in our clinic from November 2008 to November 2011, were recruited for this study. All operations were performed under general anaesthesia. Table 1 summarizes patient demographic information. Age range was 5–80 years, weight range was 17–105 kg, height range was 108–190 cm, and body mass index (BMI) range was 13.6-34.3. No patient had a history of vascular disease. Surgical procedures were most commonly open reduction with internal fixation, removal of internal fixation, external fixation, open carpal tunnel release, or arthroscopic surgery of the wrist joint (Table 2).

**Table 1:** Patients demographics

**Parameter**	**Value (mean ± standard deviation)**
Age (y)	39.8 ± 21.8
Gender (male: female)	64:256
Weight (kg)	58.5 ± 15.7
Height (cm)	159.6 ± 12.1
BMI^†^	22.7 ± 4.5
Tourniquet time (minutes)	55.2 ± 32.9
Operation time (minutes)	53.4 ± 33.0

^†^*BMI* body mass index.

**Table 2:** Surgical procedure

**Surgical procedure**	**Number of cases**
Open reduction with internal fixation	40
Removal of internal fixation	24
External fixation (radius)	11
Open carpal tunnel release	11
Arthroscopic surgery (wrist joint)	9
Percutaneous pinning (phalanx or humerus)	6
Ligament suture (interphalangeal or metacarpophalangeal joint)	3
Neurorrhaphy/ Neurolysis	4
Tendon suture/ Tenolysis/ Tendon transfer	3
Resection of benign soft tissue tumor	3
Arthrodesis for carpometacarpal joint of thumb	2
Resection of benign bone tumor (metacarpus)	1
Radial shortening for Kienbock disease	1
Ulnar shortening for ulnar abutment syndrome	1
Salvage for the pseudoarthrosis of scaphoid	1
Total	120

Approval was given by the institutional review board for the investigation in this study. And all patients signed a consent form that included a description of the protocol and potential tourniquet-related complications.

### Quality of the bloodless field

The surgeon rated the quality of the bloodless field using the evaluation method applied by Ishii et al. [7] as poor, fair, good, or excellent, and noted any changes in the quality of the bloodless field throughout the procedure. A poor field was one in which blood obscured the view; a fair field had blood present but not significantly interfering with surgery; a good field had some blood and no interference with the procedure; and an excellent field had no blood present.

## Results

### Patients’ blood pressure and inflation pressure of the tourniquet

The average duration of tourniquet use, operation time, and general anaesthesia were 55.2 minutes (range, 10–138; standard deviation [SD], 32.9), 53.4 minutes (range, 7–153; SD, 33.0), 88.7 minutes (range, 35–170; SD, 35.8), respectively. Mean initial SBP and diastolic blood pressure (DBP) just before inflation were 96.3 ± 12.7 mmHg (range, 66–131) and 51.1 ± 12.1 mmHg (range, 25–92), respectively. Table 3 summarizes tourniquet conditions. Initial tourniquet pressure (TP) range was 166–231 mmHg. Maximum and minimum TP ranges were 200–288 mmHg and 137–250 mmHg, respectively. Maximum change of SBP and DBP during the duration of tourniquet use ranged from 5–103 mmHg and 1–58 mmHg, respectively. Maximum change of SBP during a 2.5-minute interval ranged from 3–59 mmHg. In 27 patients, SBP varied greater than 40 mmHg during surgery (Table 4). In seven cases, there was a sharp rise (more than 30 mmHg) during a 2.5-minute interval (Table 5). No operation was interrupted by drastic change in SBP and no surgical fields were contaminated by oozing. SBP showed a tendency to be higher after inflation than before in 46 cases, and to be lower in 22 cases.

**Table 3:** Tourniquet conditions in this study

**Patients (N = 120)**	**Value (mean ± SD)**
initial TP^†^	196.3 ± 12.7 mmHg
maximum TP through surgery	221.7 ± 22.3 mmHg
minimum TP through surgery	191.1 ± 14.1 mmHg
maximum change of systolic BP^††^ during surgery	30.4 ± 19.4 mmHg
maximum change of diastolic BP during surgery	19.7 ± 12.0 mmHg
maximum change of systolic BP in each measurement (2.5 minutes)	13.8 ± 7.9 mmHg

^†^*TP* tourniquet pressure, ^††^*BP* blood pressure, ^*^SD standard deviation.

**Table 4:** Distribution of maximum change of systolic blood pressure during surgery

	**Number of cases**		
0 ~ 9 mmHg	12	60~69 mmHg	2
10 ~ 19 mmHg	25	70 ~ 79 mmHg	3
20 ~ 29 mmHg	30	80 ~ 89 mmHg	2
30 ~ 39 mmHg	26	90 mmHg~	2
40 ~ 49 mmHg	11	Total	120
50 ~ 59 mmHg	7		

**Table 5:** Distribution of maximum change (increase) of systolic blood pressure during a 2.5-minutes interval

	**Number of cases**		
0 ~ 9 mmHg	42	60~69 mmHg	0
10 ~ 19 mmHg	58	70 ~ 79 mmHg	0
20 ~ 29 mmHg	13	80 ~ 89 mmHg	0
30 ~ 39 mmHg	6	90 mmHg~	0
40 ~ 49 mmHg	0	Total	120
50 ~ 59 mmHg	1		

### Quality of the bloodless field

An excellent bloodless field was obtained in 119 cases. One case was rated as poor; this 44-year-old woman underwent an internal screw fixation of metacarpal fracture. In this case, we had to increase additional tourniquet pressure up to 150 mmHg at 16 minutes after the first inflation because of oozing in the operative field. There was no sharp rise of SBP before this event. After increasing inflation pressure, an excellent bloodless field was maintained for 26 minutes until the cuff was deflated. This patient did not have any pertinent past medical history or drug use and showed normal pre-operative laboratory data with the exception of hyperlipidemia. Her BMI was notably high at 34.2.

### Complications

No complication, such as compartment syndrome, deep vein disorder, skin disorder, paresis, or nerve damage, occurred during or after surgery.

## Discussion

Most surgeons seem to employ inflation pressure as follows: they use modifications in certain scenarios, for example, adjustments for age, blood pressure, weight, and extremity shape and size, or they apply a set pressure of 50–150 mmHg above SBP [1].

The most distinctive feature of the new tourniquet system used in this study is that it automatically regulates the effective pressure to supply a bloodless surgical field synchronized with SBP. It responds to a sharp rise or a drop of SBP to supply an adequate pressure; we did not need to deflate and re-inflate to employ different pressures during any procedure. This contributes not only to saved time but also to safety and accuracy by preventing futile oozing and edema in the surgical field. It also prevents excessive pressure and avoids severe neurologic complication, post-operative edema, and subsequent joint contracture. For example, SBP above 200 mmHg exceeds 300 mmHg of inflation pressure, that is thought to be border value in the safely use, in the situation additional pressure is set on 100 mmHg. With patients under general anaesthesia, the anaesthesiologist monitors vital signs and manages excessive high SBP with anaesthetic changes. Ishii et al. [7] used this tourniquet for 100 consecutive foot and ankle surgeries. They reported all cases maintained an excellent operative field without measurable bleeding and there were no postoperative complications.

There are two main physiological effects in the application of a tourniquet to a limb: ischemia and local pressure on the tissue beneath the cuff. Tourniquet paralysis is now thought to arise not only from ischemia of nerve tissue distal to the compression level but also from a combined effect with mechanical nerve compression beneath the tourniquet [1,8-11]. Ochoa et al. [9] used a primate model to demonstrate mechanical deformation of nerve fibers with displacement of the nodes of Ranvier and distortion of the paranodal myelin relating to the direction and magnitude of externally applied pressure, away from the site of compression towards uncompressed tissue. Lundborg [8] showed that ischemia alone must occur for 8 to 10 hours for the walls of endoneural vessels to be injured sufficiently to produce extravascular leakage of albumin. In the compressed nerve segment, a prominent leakage of albumin from endoneural vessels occurred after four hours of cuff compression. Their study suggested that localized compression of the nerve segment is also a principal factor in the pathogenesis of tourniquet paralysis.

In many reported cases of neurologic complications caused by pneumatic tourniquet, the pressure was too high, or applied for too long [2,3,5]. A survey of 151 members of the Australian Orthopaedic Association [3] reported the incidence of nerve palsy in the upper limb after the use of a tourniquet to be 1:5000. These nerve palsies occurred both with the use of the pressure-retained pneumatic cuff and the outmoded use of Esmarch rubber bandage as a tourniquet. A Norwegian study [2] reported two cases of major complication with tourniquet inflating duration of 130 and 180 minutes and with acceptable pressures of 250 and 300 mmHg (resulting in an incidence of three neurologic complications in 18,465 upper extremity procedures).

The inflation pressure of a pneumatic cuff may not represent the actual pressure in the soft tissues under the cuff, and pressures vary widely from the applied pressures [6,12,13]. It is difficult to measure actual perineural pressures in the midst of surgery. Several studies applied various techniques to lower cuff pressures sufficient to produce a bloodless surgical field [7,14-19]. Many authors recommend a wide tourniquet cuff to reduce the actual pressure in the tissue under the tourniquet [14]-[17], and attention should be paid to recognize the incongruities between the shape of the limb and the tourniquet resulting in pressure concentrations [12,17]. Controlled hypotension to bring down SBP can also be used to decrease direct cuff pressure against the tissue [18]. An alternating double tourniquet technique, to change the point of compression, is another method to improve [19].

Traditional recommendations suggest parameters for maximum pressure and time limits rather than the minimal effective pressure to achieve a bloodless field. The recommended maximum safe pressure for the upper limb in standard surgical texts is 250–300 mmHg for adults [4,20,21]. Little or no consensus has been reached regarding optimum tourniquet pressure [1].

Van Roekel et al. [22] reported 200 mmHg to be adequate minimum tourniquet pressure to produce a bloodless surgical field for upper limb surgery; similarly, 250 mmHg was reported to be adequate for lower limb surgery in an average sized, normotensive patient. Levy et al. [23] studied the correlations between several potential influencing parameters and the minimal tourniquet pressure in the upper limb using Doppler stethoscope, and blood pressure showed significant correlation; their formula is tourniquet pressure = (1.68 × mean arterial pressure) + 50 mmHg. They reported mean calculated minimal effective tourniquet pressures to be predicted were well below 250-300 mmHg previously recommended. However, their studies presupposed that tourniquet pressures were constant during the operation. It is nearly impossible for patient’s blood pressure to remain constant. Sharp rises in SBP might allow blood to ooze into the operative field. On the other hand, relatively high tourniquet pressures with low SBPs produces unnecessary pressure on tissues. In our study, although seven cases showed a sharp rise in SBP (over 30 mmHg) during a 2.5-minute interval, the system was able to respond automatically and produce an excellent surgical field. Twenty-seven cases exhibited a maximum change in SBP greater than 40 mmHg; an excellent surgical field was maintained in all cases. Additionally, the system could also respond automatically to decreasing changes in SBP; 22 cases showed lower SBP during the surgery than initial pressure with no problems with the surgical fields. Based on these results, we recommend the application tourniquet pressure at 100 mmHg above SBP at the upper arm.

Skin disorders are another possible complication with the tourniquet used in this study, because repeated inflation of different pressures might cause skin and subcutaneous tissue distortion. We had no case of skin complications, although this could occur despite appropriate use of the tourniquet [1,2]. Padding beneath the tourniquet is important to decrease sheer stress at the skin surface, particularly in elderly patients with delicate skin [24].

This study has some limitations. First, we are not comparing the new tourniquet system to a standard one. Our rating system for the bloodless field also might be biased as not blinded because we used single tourniquet. It is desirable to review the merit of this system relatively in the study using several tourniquets with large number of cases. Second, we did not try the additional pressure lower than 100 mmHg on SBP, which might be sufficient to supply excellent bloodless field. A continuous effort should be made to apply lower pressure as possible.

## Conclusions

In conclusion, this new tourniquet system synchronized with SBP is a reasonable device for maintaining a bloodless surgical field in upper limb surgery. It seemed to contribute to safety by lowering tissue pressure, preventing mid- and post-surgical complication. Although the incidence of complications in tourniquet usage is fortunately very rare, surgeons should choose a more practical tourniquet, such as the system used here, for upper limb surgery.

## Competing interests

The authors declare that they have no competing interests.

## Authors’ contributions

JS designed the study, performed or assisted the operation, collected the data, analyzed the data and drafted manuscript. YI designed the study, performed or assisted the operation and approved the final manuscript. HN performed or assisted the operation and collected the data. MT performed or assisted the operation and collected the data**.** All authors read and approved the final manuscript.

## Pre-publication history

The pre-publication history for this paper can be accessed here:

http://www.biomedcentral.com/1471-2482/12/17/prepub
